# A multivalent *Plasmodium falciparum* circumsporozoite protein‐based nanoparticle malaria vaccine elicits a robust and durable antibody response against the junctional epitope and the major repeats

**DOI:** 10.1002/btm2.10514

**Published:** 2023-03-28

**Authors:** Geetanjali Pendyala, J. Mauricio Calvo‐Calle, Alberto Moreno, Ravi S. Kane

**Affiliations:** ^1^ School of Chemical & Biomolecular Engineering Georgia Institute of Technology Atlanta Georgia 30332 USA; ^2^ Department of Pathology University of Massachusetts Medical School Worcester Massachusetts 01655 USA; ^3^ Emory Vaccine Center, Emory National Primate Research Center Emory University Atlanta Georgia 30329 USA; ^4^ Division of Infectious Diseases, Department of Medicine Emory University Atlanta Georgia 30303 USA; ^5^ Wallace H. Coulter Department of Biomedical Engineering Georgia Institute of Technology Atlanta Georgia 30332 USA

**Keywords:** circumsporozoite, junctional epitope, malaria, *Plasmodium falciparum*, vaccine

## Abstract

*Plasmodium falciparum* (*Pf*) malaria continues to cause considerable morbidity and mortality worldwide. The circumsporozoite protein (CSP) is a particularly attractive candidate for designing vaccines that target sporozoites—the first vertebrate stage in a malaria infection. Current *Pf*CSP‐based vaccines, however, do not include epitopes that have recently been shown to be the target of potent neutralizing antibodies. We report the design of a SpyCatcher‐mi3‐nanoparticle‐based vaccine presenting multiple copies of a chimeric *Pf*CSP (c*Pf*CSP) antigen that incorporates these important “T1/junctional” epitopes as well as a reduced number of (NANP)_*n*_ repeats. c*Pf*CSP‐SpyCatcher‐mi3 was immunogenic in mice eliciting high and durable IgG antibody levels as well as a balanced antibody response against the T1/junctional region and the (NANP)_*n*_ repeats. Notably, the antibody concentration elicited by immunization was significantly greater than the reported protective threshold defined in a murine challenge model. Refocusing the immune response toward functionally relevant subdominant epitopes to induce a more balanced and durable immune response may enable the design of a more effective second generation *Pf*CSP‐based vaccine.

## 1 INTRODUCTION

Malaria is the most relevant parasitic disease caused by five species of the genus *Plasmodium*. Progress in implementing malaria control activities in the past decade significantly reduced morbidity and mortality. Nonetheless, in 2021, 247 million malaria cases and 619,000 malaria deaths occurred worldwide, representing an increase of 6 million cases compared to 2020.^1^ Two‐thirds of these additional cases are explained by the disruption in healthcare services due to the SARS‐CoV‐2 pandemic.^1^ *Plasmodium falciparum* (*Pf*) is the most prevalent species and is the cause of more than 90% of the mortality due to malaria in humans.^2^ Therefore, there is an imminent need for an effective vaccine that protects from *Pf* infections.

The malaria parasite life cycle involves two hosts—an *Anopheles* mosquito vector and a susceptible vertebrate. When an infected female *Anopheles* mosquito draws blood from a mammalian host, it injects *Plasmodium* sporozoites that travel to the liver to infect hepatocytes to start the pre‐erythrocytic cycle. Sporozoites in the liver mature into hepatic schizonts releasing thousands of merozoites into the bloodstream that infect red blood cells to initiate the blood‐stage cycle. In red blood cells, merozoites transform into blood‐stage schizonts, some of which differentiate into gametocytes. Malaria symptoms and clinical complications in humans occur during the blood‐stage cycle. The sporogonic cycle is initiated when a mosquito draws blood from a malaria‐infected host that contains gametocytes. The gametocytes fertilize to form ookinetes that can traverse the mosquito midgut and transform into oocysts, each containing thousands of sporozoites. Finally, sporozoites migrate to the mosquito's salivary glands to initiate a new infectious cycle in a susceptible host.^3^

The pre‐erythrocytic stage, involving the migration of sporozoites to the liver leading to hepatic infection, is the first stage in a malaria infection in the vertebrate and represents a population bottleneck in the life cycle.^4^ Therefore, a vaccine that targets antigens in this stage has the potential to prevent severe clinical outcomes. The circumsporozoite protein (CSP) is a particularly attractive candidate for a sporozoite‐targeting vaccine. CSP is a surface protein densely covering sporozoites and is required for hepatocyte infection.^5^, ^6^ Due to these reasons, several attempts have been made to design a CSP‐based malaria vaccine.^7^, ^8^ CSP consists of three domains: an N‐terminal domain (NTD), the repeat region that comprises repetitive units unique to each malaria parasite, and a C‐terminal thrombospondin‐like type‐I repeat (TSR) domain.^6^ In *Pf*CSP, the repeat region consists of 35–41 NANP tetramers. In the 3D7/NF54 *Pf* isolate, the prototypical *Pf* malaria strain, the CSP repeat consists of 37 NANP tetramers (major repeats) and three alternating NVDP and NANP tetramers (minor repeats).

*Pf*CSP‐based vaccines have come a long way due to extensive research over 30 years with multiple clinical trials.^9^ One vaccine—RTS,S in the AS01 adjuvant system—has been approved for use by the World Health Organization (WHO).^1^ This vaccine is expressed in *Saccharomyces cerevisiae* and consists of 19 NANP repeats followed by the carboxy terminus of *Pf*CSP fused to the Hepatitis B surface antigen (HBsAg). The fused protein is expressed along with free HBsAg^10^ and the proteins spontaneously assemble upon cell lysis into RTS,S particles composed of a 1:4 ratio of *Pf*CSP to HBsAg.^11^, ^12^ This vaccine induces high but short‐lived antibody responses that wane after a year of administration with an efficacy of 30%–50% within the first 12 months of administration.^13^, ^14^

Collins et al.^11^ developed a RTS,S‐like vaccine (R21) that uses the *Pf*CSP‐HBsAg fusion protein as the antigen without any free HBsAg so that the *Pf*CSP to HBsAg ratio increases to 1:1. R21 administered with a saponin‐based Matrix‐M adjuvant has been tested in a phase 2 clinical trial and was found to have over 75% efficacy after a primary series of three immunizations.^15^ Importantly, R21 has shown similar levels of protection when a booster immunization is used 1 year after the initial immunization regimen^16^ suggesting that changes in the vaccine delivery system and increasing the density or exposure of relevant antigens have a significant impact on the outcome. However, current *Pf*CSP‐based vaccines do not include highly relevant epitopes upstream from the coding region of the RTS,S/R21 vaccines that are the target of protective neutralizing antibodies, supporting the concept that these vaccines can be further improved.^9^

The protective antibodies elicited by RTS,S vaccines primarily target the (NANP)_*n*_ repeat region of *Pf*CSP. Recent studies, however, have identified a junctional epitope consisting of alternating NVDP and NANP repeats that is present between the N‐terminal region and the (NANP)_*n*_ repeats in *Pf*CSP and is a target of neutralizing antibodies.^17^, ^18^, ^19^, ^20^, ^21^ Recent studies by Tan et al.^22^ and Kisalu et al.^23^ suggested that dual‐specific antibodies targeting both this junctional epitope and the (NANP)_*n*_ repeat region are more potent than the antibodies targeting only (NANP)_*n*_. Results from immunization studies conducted with junctional epitope‐based vaccines suggest the potential value of including this epitope in a *Pf*CSP‐based vaccine.^21^, ^24^ Calvo‐Calle et al.^21^, ^24^ immunized mice with a T1 (DPNANPNVDPNANPNV)‐based vaccine, that contains two copies of the junctional epitopes DPNANP, NPN, and NPNV, and found higher neutralizing activity as compared to a (NANP)_*n*_ repeat‐based vaccine. Jelínková et al.^24^ used the Qβ virus‐like particle (VLP) platform to develop a junctional epitope‐based vaccine and reported high antibody titers and long‐lasting anti‐*Pf*CSP antibody responses. We have also previously evaluated the responses of subunit peptide vaccines and VLPs containing the T1 epitope.^25^, ^26^ These studies have demonstrated that these immunogens induce high antibody titers in mice, non‐human primates, and human volunteers.

**FIGURE 1:**
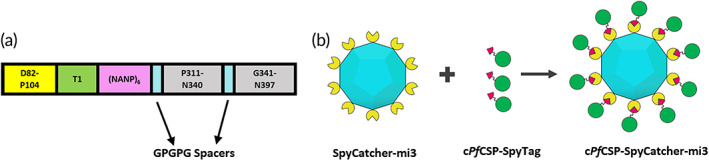
Schematic representation of c*Pf*CSP variant and assembly of c*Pf*CSP variant on SpyCatcher‐mi3. (a) c*Pf*CSP. (b) Schematic illustrating the generation of chimeric *Plasmodium falciparum* circumsporozoite (c*Pf*CSP)‐SpyCatcher‐mi3 conjugates by the reaction of c*Pf*CSP‐SpyTag variants with SpyCatcher‐mi3 nanoparticles. (c*Pf*CSP: green; SpyTag: red; SpyCatcher: yellow; mi3: cyan).

In the present work, we developed a chimeric *Pf*CSP (c*Pf*CSP) displayed multivalently on SpyCatcher‐mi3 VLPs as a strategy to generate a robust and durable immune response targeting both the T1/junctional epitopes and the (NANP)_*n*_ repeats. Based on previous studies with *Plasmodium vivax* CSP,^27^ this c*Pf*CSP incorporated a part of the N‐terminal domain containing Region I followed by a synthetic epitope based on the junctional epitope (T1) and six NANP repeats. Considering that the antibody response to *Pf*CSP repeats is restricted to a handful of MHC class II molecules^5^, ^28^, ^29^, ^30^ and that CD4+ and CD8+ T cells mediate protection against *Plasmodium* challenge by helper^26^, ^29^, ^31^, ^32^ and effector mechanisms,^33^, ^34^ our construct also included a modified C‐terminal domain containing relevant T cell epitopes to induce broader antibody responses and enhanced responses mediated by T cells (Figure 1a). The reduced number of NANP repeats aims to abate the reported immunodominance of this major repeat domain of *Pf*CSP.^35^ Further, we hypothesized that we could enhance the B‐cell response by displaying this version of *Pf*CSP multivalently from VLPs. We chose SpyCatcher‐mi3 VLPs for the multivalent display of antigens.^36^, ^37^, ^38^, ^39^, ^40^ These VLPs allow for the conjugation of proteins incorporating a SpyTag peptide sequence through the formation of an isopeptide bond, making them a versatile and efficient platform.^36^, ^38^, ^41^ Adding the SpyTag peptide to the C‐terminus of *cPf*CSP allowed us to conjugate multiple copies of *cPf*CSP to SpyCatcher‐mi3 (Figure 1b). We immunized mice with *cPf*CSP‐SpyCatcher‐mi3 using a simplified two dose regimen and characterized the elicited antibody response. *cPf*CSP‐SpyCatcher‐mi3 elicited a balanced response with high antibody titers against both the junctional epitope and the (NANP)_*n*_ repeats. Importantly, the antibody response was durable, with high antibody levels maintained for more than 9 months after the second immunization.

## 2 RESULTS AND DISCUSSION

**FIGURE 2:**
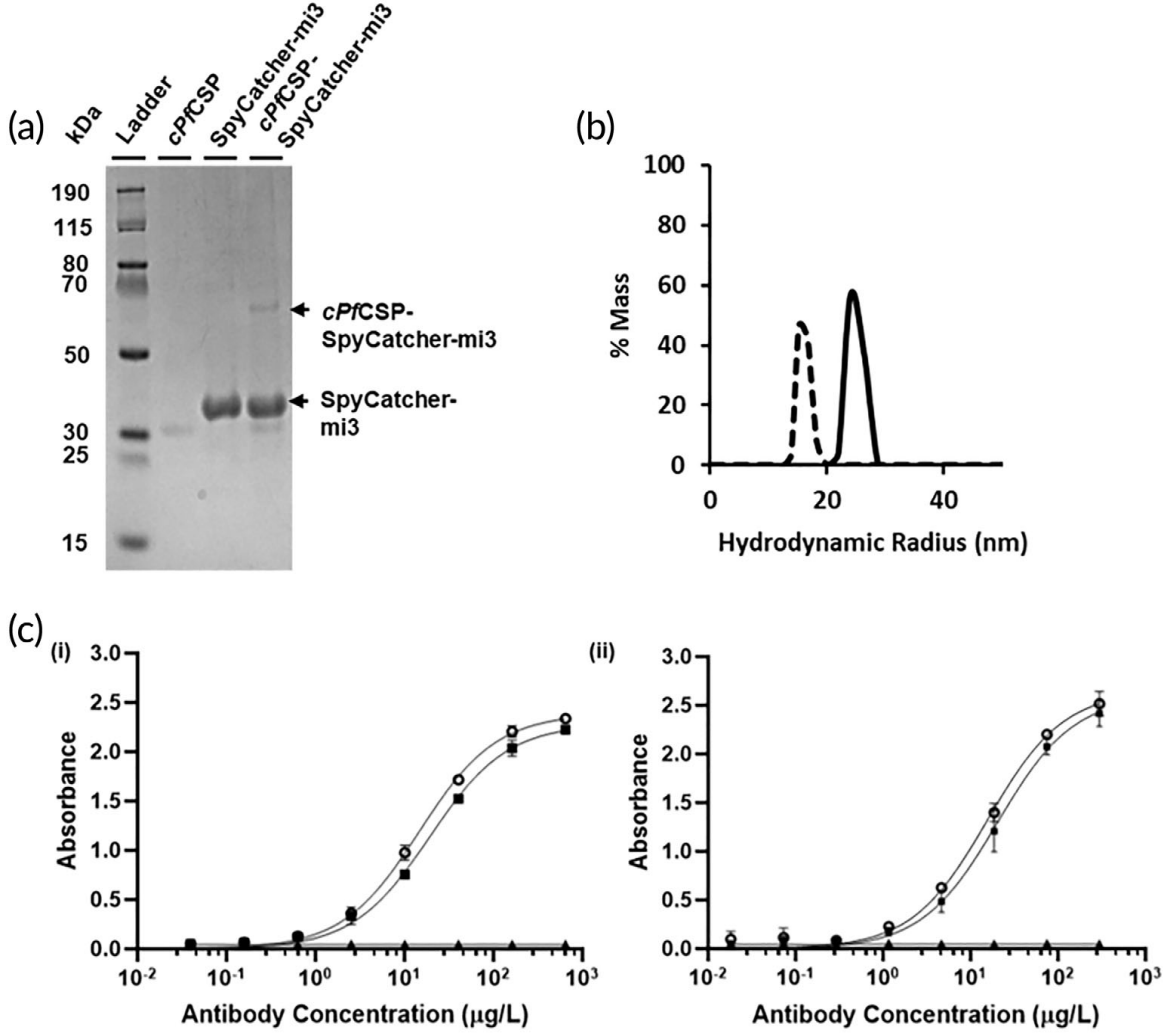
Characterization of chimeric *Pf*CSP and c*Pf*CSP‐SpyCatcher‐mi3. (a) Characterization of SpyCatcher‐mi3, c*Pf*CSP, and c*Pf*CSP‐SpyCatcher‐mi3 by SDS‐PAGE. (b) Characterization of the c*Pf*CSP‐SpyCatcher‐mi3 (solid line) and SpyCatcher‐mi3 (dashed line) by dynamic light scattering (DLS). (c) Characterization of the binding of (i) CIS43 antibody^23^ (PDB:6B5M) and (ii) 2A10 antibody^42^ (PDB: 5T0Y) to c*Pf*CSP (○), c*Pf*CSP‐SpyCatcher‐mi3 (■), and BSA (control) (▲) by enzyme‐linked immunosorbent assay (ELISA) (mean ± SD, *n* = 2: one assay with two technical replicates).

We expressed *cPf*CSP, having a SpyTag sequence followed by a 6x‐His tag at the C‐terminus in *Escherichia coli*. The 6x‐His tag enabled the purification of *cPf*CSP by immobilized metal affinity chromatography (IMAC). Subsequent purification by size exclusion chromatography (SEC) yielded pure protein as characterized by SDS‐polyacrylamide gel electrophoresis (SDS‐PAGE; Figure 2a). The band at ~30 kDa in the second lane corresponds to *cPf*CSP. Though the molecular weight of *cPf*CSP is 21 kDa, the band appears higher on a gel as reported previously.^43^

Next, we generated SpyCatcher‐mi3 nanoparticles—self‐assembled 60‐mer virus‐like particles based on a mutated aldolase protein from a thermophilic bacterium^36^ that is fused to the SpyCatcher protein. SpyCatcher‐mi3 was expressed in BL21 (DE3) competent *E. coli* cells. After cell lysis, Spycatcher‐mi3 was purified using a CaptureSelect C‐tag affinity column followed by size exclusion chromatography (SEC). Purity was assessed by SDS‐PAGE (Figure 2a).

*cPf*CSP and SpyCatcher‐mi3 were next allowed to react overnight and were characterized by SDS‐PAGE (Figure 2a). The band in the third lane of the gel at ~35 kDa corresponds to SpyCatcher‐mi3. The topmost band at ~65 kDa in the fourth lane corresponds to the *cPf*CSP‐SpyCatcher‐mi3 conjugated product. We estimated that there were 3.2 c*Pf*CSP molecules per SpyCatcher‐mi3 nanoparticle. We further characterized the conjugated product by dynamic light scattering (DLS). Figure 2b shows the comparison of particle sizes of SpyCatcher‐mi3 alone and *cPf*CSP‐SpyCatcher‐mi3. DLS indicated that the diameter of *cPf*CSP‐SpyCatcher‐mi3 nanoparticles was 48 nm, as compared to 34 nm for SpyCatcher‐mi3, confirming that the SpyTag‐SpyCatcher reaction was successful.

Next, to verify that the *cPf*CSP and *cPf*CSP‐SpyCatcher‐mi3 retained their antigenicity, we characterized the binding of *cPf*CSP and the conjugated product to dual‐specific antibodies that bind to both the T1/junctional region and the (NANP)_*n*_ repeats by an enzyme‐linked immunosorbent assay (ELISA). Figure 2c shows the successful recognition of *cPf*CSP and *cPf*CSP‐SpyCatcher‐mi3 by the CIS43^23^ (PDB: 6B5M) and 2A10^42^ (PDB: 5T0Y) antibodies.

**FIGURE 3:**
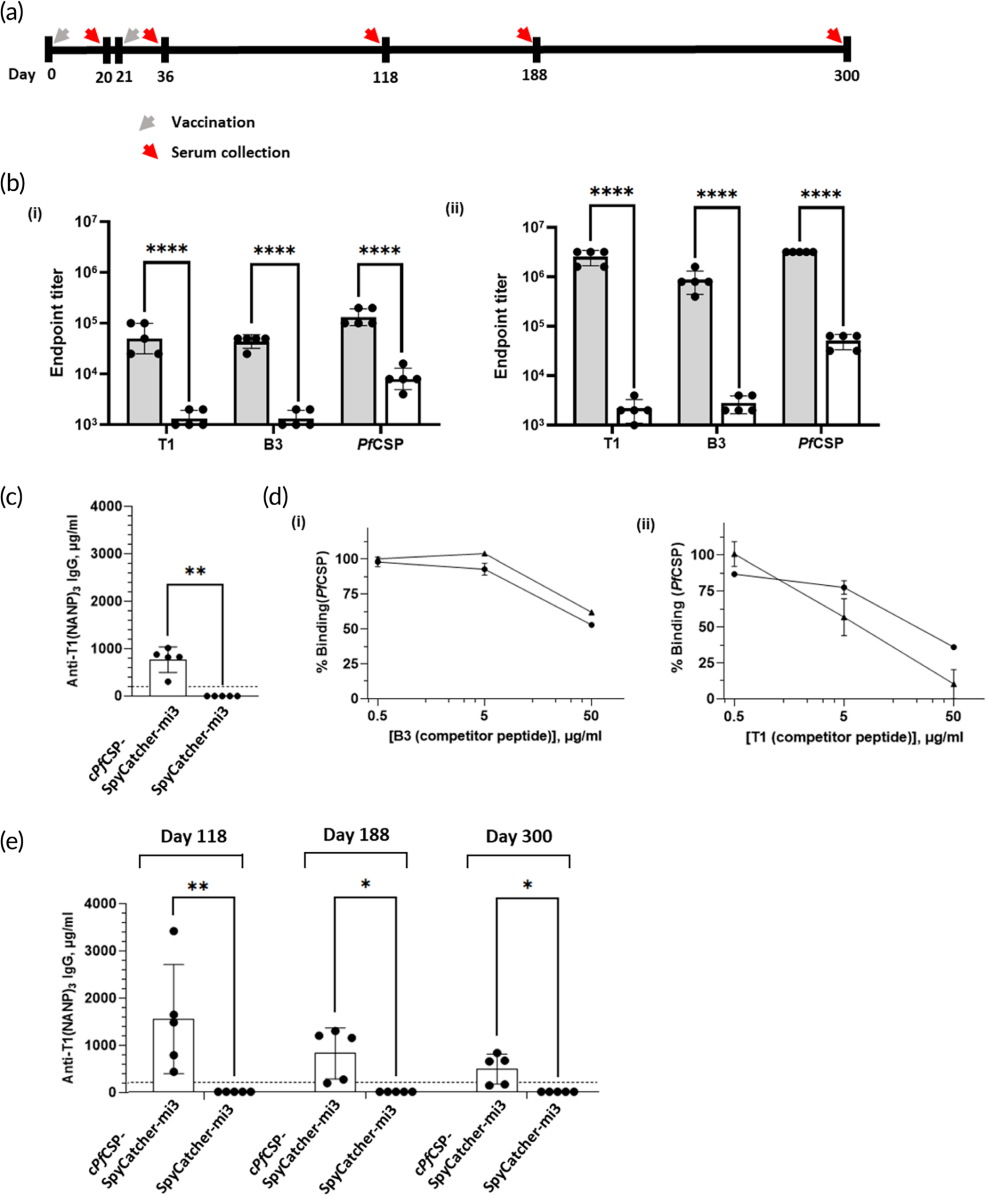
Characterization of immunogenicity elicited by c*Pf*CSP‐SpyCatcher‐mi3. (a) Schedule for mice vaccination and serum collection. (b) Antibody endpoint titers of sera from mice immunized with c*Pf*CSP‐SpyCatcher‐mi3 (gray), or SpyCatcher‐mi3 (white) (geometric mean with geometric SD, *n* = 5) (i) after the first immunization (day 20) and (ii) after the second immunization (day 36) against peptides T1, B3 ((NANP)_3_), and *Pf*CSP: one assay with sera from 5 mice; *n* = 5. ****P < 0.0001, determined by unpaired *t* test for (i) and, unpaired *t* test (against T1 and B3), and Welch's *t* test (against *Pf*CSP) for (ii). (c) Anti‐T1(NANP)_3_ IgG antibody concentration in mice immunized with c*Pf*CSP‐SpyCatcher‐mi3 was determined by comparison to a standard curve generated with the mAb 2A10; sera from mice immunized with SpyCatcher‐mi3 alone were used as control. The broken line indicates the protective anti‐repeat antibody level reported to confer protection in murine models: one assay with sera from 5 mice; *n* = 5; **P < 0.01, determined by Mann–Whitney test. (d) Characterization of antibody specificity. 5 ng/mL mAb 2A10 (▲) or a pool of sera collected from mice immunized with SpyCatcher‐mi3‐c*Pf*CSP diluted at 1:60,000 (●) were pre‐incubated with three different concentrations (0.5, 5, and 50 μg/mL) of (i) (NANP)_3_ (B3) and (ii) T1 competitor peptides and then allowed to bind to *Pf*CSP. The intensity was normalized relative to that in the absence of competing peptides. (e) Anti‐T1(NANP)_3_ IgG antibody concentration in mice 118, 188 and 300 days after first immunization with c*Pf*CSP‐SpyCatcher‐mi3 or SpyCatcher‐mi3 (control) was determined by comparison to a standard curve generated with the mAb 2A10. The broken line indicates the protective anti‐repeat antibody level reported to confer protection in murine models; one assay with sera from 5 mice; *n* = 5; *P < 0.05, determined by Welch's *t* test; **P < 0.01, determined by Mann–Whitney test at 118 days, and Welch's *t* test for 188 and 300 days.

We next characterized the immunogenicity of *cPf*CSP‐SpyCatcher‐mi3 in mice. We subcutaneously inoculated mice (*n* = 5) with *cPf*CSP‐SpyCatcher‐mi3 or SpyCatcher‐mi3 alone (control), each mixed with an equal volume of AddaVax, a vaccine adjuvant consisting of a squalene‐based oil‐in‐water nano‐emulsion. Addavax is similar to MF59, which has been licensed for use in humans,^44^, ^45^ and we have used it successfully in our previous work.^37^, ^46^, ^47^, ^48^ A boost was carried out 21 days later. Mice were bled 20 days after the initial immunization to characterize the immune response. The schedule for mice vaccination and serum collection is shown in Figure 3a. The collected sera were tested against two synthetic peptides—the T1 peptide and a peptide composed of three NANP repeats (B3)—as well as a full‐length recombinant *Pf*CSP expressed in *E. coli*. After a single immunization with *cPf*CSP‐SpyCatcher‐mi3, IgG antibody titers against *Pf*CSP had a geometric mean titer (GMT) of ~132,000 that increased by more than 24‐fold after the boosting immunization to 3,200,000 (Figure 3b). Consistent with high antibody titers against the recombinant protein, high anti‐peptide IgG antibody titers were also elicited. GMT against T1 rose more than 48‐fold, from 50,000 to 2,425,147, and GMT against B3 increased more than 18‐fold, from ~43,500 to 800,000 (Figure 3b).

It has been reported that a 200 μg/mL concentration of the monoclonal antibody 2A10, which has been reported to recognize both (NANP)_*n*_ and T1,^21^ protected a majority of mice against experimental infection with a transgenic rodent malaria parasite expressing the full‐length *Pf*CSP.^49^ Although differences in this protective serum concentration threshold of 2A10 have been described,^23^ suggesting that it is dependent on the parasite–host combination used, we decided to calibrate the antibody response elicited by immunization with *cPf*CSP‐SpyCatcher‐mi3 by estimating the levels of anti‐repeat region antibodies using a standard curve that was generated using serial dilution with the 2A10 antibody. Immunization with *cPf*CSP‐SpyCatcher‐mi3 induced high anti‐repeat antibody concentrations that ranged between 305 and 1014 μg/mL, suggesting the induction of high titers of sporozoite‐neutralizing antibodies (Figure 3c).^49^

We also used an ELISA competition assay to characterize the specificity of antibodies elicited by immunization with *cPf*CSP‐SpyCatcher‐mi3. The (NANP)_3_ peptide inhibited close to 50% of the serum binding to *Pf*CSP at 50 μg/mL concentration (Figure 3d). The T1 peptide inhibited greater than 60% of the binding of the serum to the recombinant *Pf*CSP at 50 μg/mL (Figure 3d). As expected, both synthetic peptides also inhibited the binding of 2A10 antibody to *Pf*CSP (Figure 3d). These results suggest that the immunization has elicited the desired balanced response targeting both the T1/junctional and (NANP)_*n*_ epitopes within *Pf*CSP.

Finally, to characterize the longevity of the antibody response elicited by immunization with SpyCatcher‐mi3‐c*Pf*CSP, we estimated the levels of anti‐repeat region antibodies in sera 118, 188, and 300 days after the first immunization (i.e., 97, 167, and 279 days after the booster immunization). As seen in Figure 3e, anti‐repeat antibody levels were maintained above the protective threshold^49^ even on day 300, more than 9 months after the booster immunization. We note that there is variation in anti‐repeat antibody levels from mouse to mouse. We have consistently observed variations in the antibody responses elicited by malaria vaccines in animal models and humans.^25^, ^28^, ^50^ Collectively, the data show that immunization with SpyCatcher‐mi3‐c*Pf*CSP elicited balanced, potent, and long‐lasting antibody responses against the relevant target epitopes in *Pf*CSP.

## 3 CONCLUSIONS

In this study, we have successfully designed and produced multivalent *cPf*CSP‐SpyCatcher‐mi3 nanoparticles. The chimeric multivalent vaccine construct incorporates the T1/junctional region, an epitope not included in RTS,S or R21 vaccines, that is the target of potent neutralizing antibodies. *cPf*CSP‐SpyCatcher‐mi3 also includes a reduced number of (NANP)_*n*_ repeats to reduce its described immunodominance reported as detrimental for the response to subdominant epitopes within *Pf*CSP.^35^ *cPf*CSP‐SpyCatcher‐mi3 was immunogenic in mice eliciting high and durable IgG antibody titers with a two‐immunization regimen. Moreover, immunization elicited antibody responses against the T1/junctional region as well as the (NANP)_*n*_ repeats. Importantly, the antibody concentration elicited by immunization was greater than the threshold for protection defined in a murine challenge model.^49^ These proof‐of‐principle studies demonstrate the feasibility of refocusing the immune response toward subdominant epitopes to induce a more balanced and durable immune response relevant to developing a more effective second generation of *Pf*CSP‐based vaccines.

## 4 METHODS

### 4.1 Design of the c*Pf*CSP

c*Pf*CSP was designed using the 3D7/NF54 sequence (ID: Q7K740)^51^ following a similar approach that we used to design and express a recombinant chimeric *P. vivax* CSP (c*Pv*CSP).^27^ The protein incorporates (Figure 1a): (1) The segment D_82_‐P_104_ recognized by the protective monoclonal antibody 5D5^52^ that includes the proteolytic cleavage site^17^, ^18^ and Region I (RI) and the stretch of positively charged residues upstream of RI, which contain the binding domain to heparan sulfate involved in the attachment of sporozoites to hepatocytes.^53^, ^54^ (2) The junctional domain T1, recognized by the protective monoclonal antibody CIS43.^23^ (3) (NANP)_6_ representing the major repeat region with a reduced copy number to avoid the reported induction of short‐lived plasmablasts since the native protein with a high copy number of repeats acts as a T cell‐independent antigen.^55^ (4) The immunodominant Th2R/T* (P_311_‐N_340_), Th3R, and a C‐terminal T cell epitope mapped in murine and human models (G_341_‐N_397_)^56^, ^57^ separated by GPGPG spacers as we have described with other *Plasmodium* chimeric proteins.^27^, ^58^, ^59^, ^60^ The sequence representing Th3R is an extended version that includes the H‐2K^k^ restricted CTL epitope DYENDIEKKI (D_359_‐I_368_).^61^, ^62^

### 4.2 Cloning of c*Pf*CSP constructs, anti‐CSP antibodies, and SpyCatcher‐mi3

#### 4.2.1 c*Pf*CSP

The gene encoding *cPf*CSP followed by a GPGPG spacer, a SpyTag, and a 6x His‐Tag was optimized for bacterial expression, synthesized, and cloned into the pET‐28b vector by Gene Universal Inc. (Newark, DE). The synthesized plasmid was transformed into SHuffle® T7 Competent *E. coli* cells (New England Biolabs) which were then incubated overnight at 30°C in the presence of kanamycin (50 mg/L). The cells were then stored at −80°C in 25% glycerol for future use.

#### 4.2.2 Anti‐CSP antibodies

Genes encoding the light and heavy chains of the CIS43^23^ (PDB: 6B5M) and 2A10^42^ (PDB: 5T0Y) antibodies were optimized for mammalian expression. The light chains were synthesized and cloned into the TGEX‐LC vector. The heavy chains for CIS43 and 2A10 were cloned into TGEX‐HC vector by Gene Universal Inc. (Newark, DE). All the plasmids were transformed into 5‐alpha competent *E. coli* cells (New England Biolabs). Transformed cells were grown overnight at 37°C in 100 mL of 2xYT medium in the presence of ampicillin (100 mg/mL). The DNA was then extracted and purified from the transformed cells using E.Z.N.A Plasmid Maxi Kit (Omega). The amplified DNA was stored for future use.

#### 4.2.3 SpyCatcher‐mi3

The gene encoding the SpyCatcher‐mi3 fusion protein^36^ was optimized for bacterial expression, synthesized, and cloned into the pET‐21a vector, by Gene Universal Inc. (Newark, DE). The plasmid was transformed into BL21(DE3) competent *E. coli* cells (New England Biolabs), and the transformed cells were grown overnight at 37°C in the presence of kanamycin (50 mg/L). The cells were stored at −80°C in 25% glycerol for future use. All transformations were performed according to manufacturer protocols.

#### 4.2.4 Expression and purification of c*Pf*CSP

A 5 mL starter culture was grown for 12–16 h from a glycerol stock of cells transformed with the DNA encoding *cPf*CSP. The starter culture was then added to 1 L of 2xYT medium supplemented with kanamycin and incubated at 30°C. When the OD_600_ reached 0.6, the culture was induced with 1 mM isopropyl β‐D‐1‐thiogalactopyranoside (IPTG) (Fisher Scientific), and the temperature was lowered to 16°C. The culture was incubated overnight.

The following steps were performed at 4°C. On the next day, the cells were pelleted by centrifuging at 7000*g* for 10 min. They were then resuspended in 20 mL of IMAC binding buffer (100 mM Tris, 150 mM NaCl, 20 mM imidazole, pH 8.0) containing 0.5 mg/mL lysozyme, 125 units of benzonase, and half a tablet of EDTA‐free protease inhibitor cocktail (Fisher Scientific). A 5% sodium deoxycholate solution (Alfa Aesar) was added to the cell suspension before sonicating it twice for 3 min at 30% amplitude with 3 s on and 3 s off pulses. The cell suspension was then centrifuged at 12,000*g* for 30 min to separate the supernatant containing *cPf*CSP from the cell debris.

The supernatant was then purified by Ni‐NTA affinity chromatography. The gravity‐flow columns containing 1 mL column volume (CV) of Ni‐NTA resin (Thermo Fisher Scientific) were equilibrated with 20 CVs of IMAC binding buffer. The supernatant from the previous step was then poured onto the resin. The resin was then washed with 20 CVs of wash buffer (100 mM Tris, 150 mM NaCl, 75 mM imidazole, pH 8.0), followed by elution with 10 CVs of elution buffer (100 mM Tris, 150 mM NaCl, 400 mM imidazole, pH 8.0). The eluate was concentrated in an Amicon spin filter with 10 kDa MWCO to 1 mL and was further purified by size exclusion chromatography (SEC) using a Superdex 200 Increase 10/300 GL column. Fractions corresponding to *cPf*CSP were collected and concentrated using spin filters. Pure *cPf*CSP was stored at 4°C for future use.

#### 4.2.5 Expression and Purification of Anti‐CSP antibodies

The antibodies (CIS43 and 2A10) were expressed in HEK293F cells using the manufacturer's protocol. In brief, the constructs were transfected using the ExpiFectamine™ 293 transfection kit (Gibco), and the antibodies were harvested 5 days later.

The antibody cultures were centrifuged at 7000*g* for 7 min, and the supernatant was dialyzed overnight against PBS (137 mM NaCl, 2.7 mM KCl, 8 mM Na_2_HPO_4_, and 2 mM KH_2_PO_4_). The CIS43 and 2A10 antibodies were purified using a MabSelect SuRe column (GE) based on the manufacturer's protocol. Purified antibodies were then concentrated using Amicon spin filters and stored in 30% glycerol at −20°C for future use.

#### 4.2.6 Expression and Purification of SpyCatcher‐mi3

A 5 mL starter culture was grown for 12–16 h from a glycerol stock of cells transformed with the DNA encoding SpyCatcher‐mi3. The starter culture was then added to 1 L of 2xYT medium supplemented with kanamycin and incubated at 37°C. When the OD_600_ reached 0.6, the culture was induced with 0.5 mM isopropyl β‐D‐1‐thiogalactopyranoside (IPTG) (Fisher Scientific), and the temperature was lowered to 22°C. The culture was incubated overnight.

The next day, the cells were pelleted by centrifuging at 7000*g* for 10 min. They were then resuspended in 20 mL of CaptureSelect Equilibration Buffer (25 mM Tris, 150 mM NaCl, pH 8.5) containing 2 mg/mL lysozyme, 125 units of benzonase, and half a tablet of EDTA‐free protease inhibitor cocktail (Fisher Scientific). The cell suspension was incubated at room temperature for 1 h, followed by sonication for 5 min at 30% amplitude with 5‐s on and 5‐s off pulses. The cell suspension was then centrifuged at 17,000*g* for 30 min to separate the supernatant containing SpyCatcher‐mi3 from the cell debris.

The supernatant was then purified using CaptureSelect C‐tag Affinity Matrix (ThermoFisher Scientific). A gravity‐flow column containing 5 mL of C‐tag Affinity Matrix was equilibrated with 10 CVs of CaptureSelect Equilibration Buffer. The supernatant from the previous step was then poured onto the matrix. The C‐tag affinity matrix was then washed with 10 CVs of equilibration buffer, followed by elution with 5 CVs of CaptureSelect Elution Buffer (20 mM Tris, 2 M MgCl_2_, pH 8.5). The eluate was concentrated in an Amicon spin filter with 10 kDa MWCO and was further purified by size exclusion chromatography (SEC) using a Superdex 200 Increase 10/300 GL column. Fractions corresponding to SpyCatcher‐mi3 were analyzed for aggregates using DLS. Fractions containing large amounts of aggregates were discarded, and the rest were stored at 4°C for future use.

### 4.3 Conjugation of cPfCSP to SpyCatcher‐mi3 nanoparticles

An optimum stoichiometric ratio for the reaction between *cPf*CSP and SpyCatcher‐mi3 was determined by setting up a series of small‐scale reactions. *cPf*CSP and SpyCatcher‐mi3 were mixed and allowed to react overnight at 4°C. The optimal ratio was determined by characterizing the reaction mixtures using SDS‐PAGE.

### 4.4 SDS‐PAGE

Protein samples were diluted in Nu‐PAGE lithium dodecyl sulfate (LDS) loading buffer (Invitrogen) to a final quantity of 0.2–0.5 μg. Fifteen microliters of protein samples and 5 μL of PageRuler Plus Prestained Protein Ladder were added to the wells of a 4%–12% Bis‐Tris gel (Thermo Scientific). Gels were run in MES‐SDS buffer at 120 V for 40 min and stained with SimplyBlue SafeStain (Invitrogen) for 15 min. The gels were then destained and imaged using the ChemiDoc MP imaging system (Bio‐Rad).

### 4.5 Dynamic light scattering

One hundred microliters samples of *cPf*CSP, SpyCatcher‐mi3, and *cPf*CSP‐SpyCatcher‐mi3 at a concentration of ~0.5 mg/mL were added to a UVette (Eppendorf). Dynamics software and a DynaPro NanoStar Dynamic Light Scattering detector were used to collect three acquisitions for each measurement. Acquisitions were averaged, and results were displayed as % Mass using the Isotropic Sphere model.

### 4.6 ELISA

To determine the immunoreactivity of the proteins by ELISA, the wells of a 96‐well plate were coated with 0.1 μg of protein suspended in 100 μL of PBS and incubated at room temperature for 1 h. The wells were then blocked with 5% (w/v) bovine serum albumin (BSA) solution in 0.1% tween‐added PBS (PBST). After incubating for 1 h and washing three times with PBST, 100 μL of the corresponding primary antibodies—CIS43 or 2A10—in PBST with 1% BSA were added to the wells. After three PBST washes following a 1 h incubation, 100 μL of the corresponding diluted secondary antibody was added to the wells. After a final 1 h incubation and wash cycle, the 96‐well plate was developed by adding 100 μL of 3,3′,5,5′‐tetramethylbenzidine (TMB) substrate. After 2.5 min, 100 μL of stop solution (160 mM sulfuric acid) was added, and the absorbance at 450 nm was measured.

The specificity of antibodies elicited by immunization was also determined by ELISA using Immulon 2HB ELISA plates (Thermo Scientific, Waltham, MA) coated with 1 μg/mL of the synthetic peptides T1 or B3 diluted in PBS or full‐length *Pf*CSP kindly provided by Gennova (Pune, India)^63^ diluted in carbonate buffer and incubated overnight at 4°C. The synthetic peptides used for coating have been previously described and synthesized incorporating cysteine residues at the amino and carboxyl‐terminal ends with the topology Cys‐T1‐Cys or Cys‐B3‐Cys.^58^ The wells were then blocked with PBS‐1% BSA blocking solution (SeraCare) for 2 h at 37°C. Sera samples from immunized mice were serially diluted in PBS‐0.5% BSA containing 0.05% Tween‐20 and incubated for 1 h at 37°C. Reactivity was determined using HRP‐labeled goat anti‐mouse IgG (SouthernBiotech) and SureBlue Reserve TMB substrate (SeraCare). Optical densities were determined using a 450 nm filter on a VERSAmax ELISA reader (Molecular Device Corporation, Sunnyvale, CA). The cutoff value was set at the highest dilution of sera resulting in an O.D. greater than three standard deviations (SD) above the mean obtained using sera from unimmunized mice. ELISA results are presented as the reciprocal of the endpoint dilution. To determine anti‐*Pf*CSP IgG concentration, standard curves for the monoclonal antibody 2A10 were generated using 10 twofold dilutions of the monoclonal Ab starting at 100 ng/mL. Sera samples were tested at 1:50,000, 1:100,000, and 1:200,000 dilutions. The concentrations were determined using GraphPad's four‐parameter logistic (4PL) curves. For the ELISA competition assays, sera samples were tested at 1:30,000, 1:60,000, and 1:120,000 dilutions and pre‐incubated with T1 or B3 synthetic peptides without cysteine residues at 0.5, 5, or 50 μg/mL. Samples were then tested for binding to plates coated with *Pf*CSP.

### 4.7 Mice and immunization regimens

Six‐ to eight‐week‐old female C57BL/6 mice (The Jackson Laboratory) were obtained and housed in micro‐isolation cages. All animal experiments and procedures were performed following guidelines and approved by the Emory University Institutional Animal Care and Use Committee. Mice were randomized into two groups of 5 mice each. Mice in group 1 received subcutaneous immunizations with c*Pf*CSP‐SpyCatcher‐mi3 (2.57 μg of the c*Pf*CSP per mouse), emulsified at a 1:1 ratio with Addavax adjuvant (InvivoGen, San Diego, CA) on days 0 and 21. Mice in group 2 were immunized with the 33.9 μg of SpyCatcher‐mi3 per mouse (the same amount of SpyCatcher‐mi3 as in group 1), also emulsified at a 1:1 ratio with Addavax. Sera samples were collected 24 h before each immunization, and 36, 118, 188, and 300 days after the first immunization to determine antibody responses. Antibody responses were assessed as total IgG antibody titers and determined by ELISA as described.^59^

## AUTHOR CONTRIBUTIONS

**Geetanjali Pendyala:** Data curation (equal); formal analysis (equal); investigation (equal); writing – original draft (equal); writing – review and editing (equal). **J. Mauricio Calvo‐Calle:** Writing – review and editing (equal). **Alberto Moreno:** Conceptualization (equal); data curation (equal); formal analysis (equal); investigation (equal); writing – review and editing (equal). **Ravi S. Kane:** Conceptualization (equal); supervision (equal); writing – original draft (equal); writing – review and editing (equal).

## CONFLICT OF INTEREST STATEMENT

The authors declare no conflicts of interest.

## Data Availability

The data that support the findings of this study are available from the corresponding authors upon reasonable request.
